# Relationship of Blood Mercury Levels to Health Parameters in the Loggerhead Sea Turtle (*Caretta caretta*)

**DOI:** 10.1289/ehp.9918

**Published:** 2007-07-11

**Authors:** Rusty D. Day, Al L. Segars, Michael D. Arendt, A. Michelle Lee, Margie M. Peden-Adams

**Affiliations:** 1 National Institute of Standards and Technology, Hollings Marine Laboratory, Charleston, South Carolina, USA; 2 College of Charleston, Grice Marine Laboratory, Charleston, South Carolina, USA; 3 South Carolina Department of Natural Resources, Marine Resources Division, Charleston, South Carolina, USA; 4 Department of Pediatrics, Medical University of South Carolina, Charleston, South Carolina, USA; 5 Marine Biomedicine and Environmental Science Center, Medical University of South Carolina, Charleston, South Carolina, USA; 6 Mystic Aquarium and Institute for Exploration, Mystic, Connecticut, USA

**Keywords:** aspartate aminotransferase (AST), blood, creatine phosphokinase (CPK), hematocrit, immunotoxicity, lymphocytes, loggerhead sea turtles, mercury, methylmercury, toxicity

## Abstract

**Background:**

Mercury is a pervasive environmental pollutant whose toxic effects have not been studied in sea turtles in spite of their threatened status and evidence of immunosuppression in diseased populations.

**Objectives:**

In the present study we investigate mercury toxicity in loggerhead sea turtles (*Caretta caretta*) by examining trends between blood mercury concentrations and various health parameters.

**Methods:**

Blood was collected from free-ranging turtles, and correlations between blood mercury concentrations and plasma chemistries, complete blood counts, lysozyme, and lymphocyte proliferation were examined. Lymphocytes were also harvested from free-ranging turtles and exposed *in vitro* to methylmercury to assess proliferative responses.

**Results:**

Blood mercury concentrations were positively correlated with hematocrit and creatine phosphokinase activity, and negatively correlated with lymphocyte cell counts and aspartate amino-transferase. *Ex vivo* negative correlations between blood mercury concentrations and B-cell proliferation were observed in 2001 and 2003 under optimal assay conditions. *In vitro* exposure of peripheral blood leukocytes to methylmercury resulted in suppression of proliferative responses for B cells (0.1 μg/g and 0.35 μg/g) and T cells (0.7 μg/g).

**Conclusions:**

The positive correlation between blood mercury concentration and hematocrit reflects the higher affinity of mercury species for erythrocytes than plasma, and demonstrates the importance of measuring hematocrit when analyzing whole blood for mercury. *In vitro* immunosuppression occurred at methylmercury concentrations that correspond to approximately 5% of the individuals captured in the wild. This observation and the negative correlation found *ex vivo* between mercury and lymphocyte numbers and mercury and B-cell proliferative responses suggests that subtle negative impacts of mercury on sea turtle immune function are possible at concentrations observed in the wild.

Mercury has been identified as one of the most serious environmental threats to the well-being of wildlife in the southeastern United States (Facemire et al. 1995). The prevalence of Hg in aquatic species has also prompted concerns for the health of subsistence fishermen and the general population who regularly consume fish. This concern is evident in the fact that 76% of fish consumption advisories in the United States in 2003 were due at least in part to Hg, for a total of 5,289,020 hectares of lakes and 1,234,127 river miles [U.S. Environmental Protection Agency (EPA) 2004]. The toxic effects of Hg have been demonstrated in mammals, birds, and fish and include neurotoxicity, impaired growth and development, reduced reproductive success, liver and kidney damage, and immunomodulation (Wiener et al. 2003; Zelikoff et al. 1994).

Reptiles are prominent members of ecosystems and often have life history characteristics that make them vulnerable to Hg accumulation (e.g., long life span, high trophic level, aquatic habitat). Despite the fact that many of these reptilian species also have a tenuous conservation status, there are currently few data on the toxicity of Hg in this taxon. One such example is the sea turtle, of which all species are classified as either threatened or endangered. Many anthropogenic factors have been implicated in the decline in sea turtle populations, including directed harvest for food and trade, fisheries bycatch, and degradation of nesting beach habitat (Lutcavage et al. 1997). However, the role of chemical pollutants in marine turtle health is largely unknown. It is therefore important to understand the risk that contaminants pose to the general health and immunologic function of sea turtles because these effects could also impact the survival of their populations.

Several studies have measured Hg levels in tissues from juvenile and adult sea turtles (Anan et al. 2001; Davenport and Wrench 1990; Day et al. 2005; Godley et al. 1999; Gordon et al. 1998; Maffucci et al. 2005; Orvik 1997; Presti 1999; Sakai et al. 1995, 2000a, 2000b; Storelli et al. 1998, 2005; Wang 2005). However, relatively few studies have assessed health parameters in sea turtles in relation to environmental contaminants (Keller et al. 2004, 2005, 2006a, 2006b; Lutcavage et al. 1995; Peden-Adams et al. 2002, 2003; Podreka et al. 1998), and these studies have focused primarily on organic contaminants rather than metals. Balazs and Pooley (1991) suggested that environmental contaminants are a possible factor contributing to the development of the viral disease fibropapillomatosis in sea turtles by reducing immune function. Some locations in Florida (Indian River Lagoon and Florida Bay) exhibit up to 70% prevalence of this disease, and these environments also have elevated levels of Hg (Ache et al. 2000; Cantillo et al. 1999; Trocine and Trefry 1996). In the present study we investigated the relationship of blood Hg levels in loggerhead sea turtles (*Caretta caretta*) off the coasts of South Carolina, Georgia, and northern Florida to various immunologic and general health parameters. Using an integrated approach, correlative field data collection from wild-caught loggerhead sea turtles were combined with laboratory *in vitro* exposure of loggerhead lymphocytes to methylmercury (MeHg).

## Material and Methods

### Sample collection and processing

**Figure 1:**
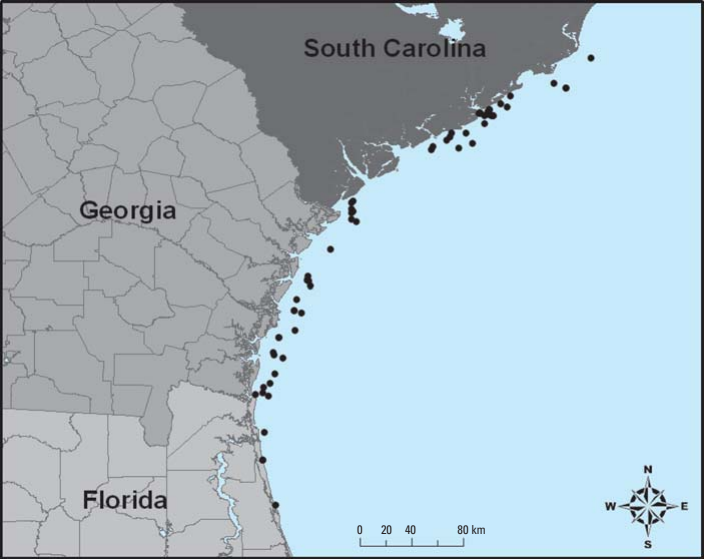
Map showing stations where loggerhead sea turtles were captured and sampled for clinical blood chemistry profiles, immune function parameters, or blood mercury concentration.

Free-ranging subadult and adult loggerhead sea turtles with straight carapace lengths (measured from the nuchal notch to the most posterior marginal notch) between 51.4 and 94.9 cm were captured as part of a sea turtle index-of-abundance study conducted by the South Carolina Department of Natural Resources (National Marine Fisheries Service Scientific Research Permit No. 1245). All samples for the present study were collected in 2001 and 2003 between the last week in May and the last week in July. Turtles were captured using 20.3-cm stretch mesh trawl nets without turtle excluder devices and a trawl tow time of 30 min. Approximately 750 trawl stations were randomly selected each year and sampled from a sampling “universe” representing every 1 square nautical mile in water depths of 4.8–14.9 m between Winyah Bay, South Carolina, and St. Augustine, Florida (Figure 1). Turtles were tagged, measured, weighed, and released near their capture location. Blood was collected from the dorsocervical sinus using double-ended Vacutainer needles directly into heparinized Vacutainer blood collection tubes (BD, Franklin Lakes, NJ) and kept cool until processing. Samples for blood chemistry panels, differential white blood cell (WBC) counts, and lymphocyte proliferation were analyzed within 36 hr of collection; samples for plasma lysozyme activity and Hg determination were stored at –20°C until analysis. All animals in this study were treated humanely and with regard for alleviation of suffering.

### Total Hg analysis

We determined total Hg (THg) concentration (based on wet mass) in tissues using isotope dilution cold vapor inductively coupled plasma mass spectrometry (ICPMS) at the National Institute of Standards and Technology (NIST, Hollings Marine Laboratory, Charleston, SC). This analytical method has been previously described in detail (Christopher et al. 2001). Briefly, isotopically enriched ^201^Hg spike solution was prepared and calibrated using NIST Standard Reference Material (SRM) 3133 Hg Spectrometric Solution. The spike was then added quantitatively to approximately 0.8 g blood to yield an isotopic ratio (^201^Hg/^202^Hg) that minimizes random error propagation. Samples were then digested and equilibrated in a PerkinElmer Multiwave microwave oven (PerkinElmer, Shelton, CT) at the highest possible temperatures (up to 300°C) and pressures (up to 8 MPa) using quartz microwave decomposition vessels and high-purity nitric acid (Fisher Scientific, Suwanee, GA). The digestant was mixed with a tin chloride and hydrochloric acid reductant solution in a gas–liquid separator, allowing cold vapor transfer of the resulting Hg^0^ in a stream of argon to the ICPMS injector. We used a Plasma Quad 3 ICPMS (VG Elemental, Windsford, Cheshire, UK) using typical ICP power and gas flows in time-resolved analysis mode for measurement of isotopic ratios.

### Clinical chemistry and complete blood counts

Blood samples were collected for plasma chemistry and complete blood count (CBC) analysis (according to the contract laboratory’s specifications) and were shipped overnight on cold packs to Antech Diagnostics (Memphis, TN) for a complete reptilian profile according to their “test express” option so that all samples were analyzed by the same laboratory, and theoretically, by the same technician. Therefore 6 samples collected in 2001 were combined with 22 samples from 2003 for statistical analysis. Samples for clinical chemistries were collected in heparinized serum separator microtainers and CBC analyses, including WBC differentials and estimated WBC counts, were conducted on blood smears from heparinized whole blood.

### *Lymphocyte proliferation: *ex vivo *THg exposure*

The proliferative response was measured using optimized methods described by Keller et al. (2005, 2006a). Briefly, peripheral blood leukocytes (PBLs) were isolated by a slow spin technique within 36 hr of blood collection. Cells were rinsed once with RPMI 1640 media (Mediatech Inc., Herndon, VA) that was supplemented with 5% fetal bovine serum (FBS; Hyclone, Logan, UT, in 2001; Gemini, Calabasas, CA, in 2003), and final concentrations of 1% (volume fraction) of 100x solution of nonessential amino acids (Gibco, Grand Island, NY), 1 mmol sodium pyruvate (Gibco), 10 mmol HEPES (Mediatech Inc.), 50 IU/mL penicillin (Mediatech Inc.), and 50 μg/mL streptomycin (Mediatech Inc.), which was initially brought to a pH of 6.9. The change in FBS source was shown in paired samples in 2003 to not alter the lymphocyte proliferation response (Keller et al. 2005). Viable PBLs were counted by trypan blue exclusion and diluted to 1.8 × 10^6^ cells/mL in the supplemented RPMI 1640 medium. Aliquots (100 μL) of the resulting cell suspensions were dispensed into 96-well plates (1.8 × 10^5^ cells/well) containing triplicate wells of either 20 μg/mL concanavalin A (Con A, type IV-S; Sigma Chemical Co., St. Louis, MO), 5 μg/mL phytohemagglutinin (PHA; L9132; Sigma), 10 μg/mL lipopolysaccharide (LPS; *E. coli* 0111:B4; Sigma), 0.2 μg/mL, phorbol 12,13-dibutyrate (PDB; Sigma), or supplemented RPMI 1640 (unstimulated control wells). All mitogen concentrations are expressed in micrograms per milliliter of culture (final culture concentration; culture volume was 200 μL).

Cells were incubated at 30°C with 5% CO_2_ for 96 hr (LPS and PDB in 2001 and 2003) or 120 hr (Con A and PHA in 2001 and 2003; LPS and PDB in 2001). Following either the 96-hr or the 120-hr initial incubation, 0.5 μCi ^3^H-thymidine (ICN Biomedicals Inc., Irvine, CA) was added to each well in a volume of 100 μL. Plates were further incubated for 16 hr and then harvested onto Unifilter plates (Packard, Meridian, CT) using a Packard Filtermate 96-well plate harvestor, and the plates were allowed to dry. Once the plates were dry, a 25-μL aliquot of Microscint 20 (Packard) was added to each well, and the samples were analyzed using a Packard Top Count-NXT scintillation counter. The stimulation index (SI) was calculated for each sample as the counts per minute (cpm) of mitogen-stimulated cells divided by the cpm of unstimulated control (media only) cells.

### *Lymphocyte proliferation: *in vitro*MeHg exposure*

PBLs from loggerhead turtles captured and released in the summers of 2003 and 2004 were isolated and diluted as described above. Cells were plated at 1.8 × 10^5^ cells/well in a final volume of 200 μL/well. Final mitogen concentrations in the culture wells were 5 μg/mL PHA 0.2 μg/mL PDB, or supplemented RPMI 1640 (unstimulated control wells). MeHg (Sigma) was diluted in sterile tissue culture water (Mediatech Inc.), to a stock of 0.1 mg/mL, and dilutions of dosing solutions were made from this stock into supplemented RPMI 1640 media as described above. MeHg was added in a volume of 5 μL to yield the appropriate concentrations in a final well volume of 205 μL. The concentrations of MeHg in the culture wells for proliferation studies were 0, 0.01, 0.03, 0.05, 0.1, 0.35, and 0.7 μg/g. The concentrations of MeHg in the culture wells for cell viability studies were 0, 0.01, 0.03, 0.05, 0.1, 0.5, and 1.0 μg/g.

PBLs from individual turtles were exposed to all concentrations, resulting in a dose–response relationship for each individual animal. Cells were incubated at 30°C with 5% CO_2_ for 120 hr at which time ^3^H-thymidine was added; proliferation was measured 16 hr later as described above (total incubation, 136 hr). Cell viability was determined by trypan blue exclusion of cells from an additional nonstimulated replicate of each contaminant concentration at time of harvest (136 hr).

### Lysozyme activity

We measured lysozyme activity using a standard turbidity assay as previously described by Demers and Bayne (1997) with slight modifications. A 1-mg/mL stock solution of hen egg lysozyme (HEL; Sigma) was prepared in 0.1 mol/L phosphate buffer (pH 5.9) and aliquots were frozen until used. A solution of *Micrococcus lysodeikticus* (Sigma) was prepared fresh daily by dissolving 50 mg of the lyophilized cells in 0.1 mol/L phosphate buffer, pH 5.9 (100 mL). HEL was serially diluted in phosphate buffer to produce a standard curve of 40, 20, 10, 5, 2.5, 1.25, 0.6, 0.3, and 0 μg/μL. Each concentration (25 μL/well) was added to a 96-well plate in triplicate. For each sample, a 25-μL aliquot of test plasma was added in quadruplicate to the plate. The solution of *M. lysodeikticus* (175 μL/well) was added quickly to each of the three sample wells and to the standard wells. To the fourth well containing plasma, 175 μL phosphate buffer was added as a blank. Plates were assessed immediately at 450 nm, at time 0 (T_0_) and again after 5 min (T_5_) with a spectrophotometer (Packard). Absorbance unit values at T_5_ were subtracted from those at T_0_ to determine the change in absorbance. The absorbance unit value for the blank sample well was subtracted from the average of the triplicate sample wells to account for slight hemolysis in some of the samples. The resultant absorbance unit value was converted to micrograms per microliter HEL using the linear equation from the standard curve. Lysozyme assays on samples from 2001 and 2003 were performed by two different technicians; therefore, these two data sets were not combined for statistical analysis.

### Corticosterone and testosterone

We measured plasma testosterone and corticosterone levels using standard radioimmunoassays, with the same protocol for both steroid hormones. Following an ether extraction, commercially available tritiated hormone and steroid antibody were added to duplicate samples and controls. The unbound hormone was separated by dextran-coated charcoal in phosphate buffer. Tritiated hormone in the supernatant was measured using a liquid scintillation counter, and RIAMENU software (developed by Paul Licht; University of California, Berkeley, CA) was used to determine the amount of native hormone in the plasma samples. These assays are sensitive in the range of tens of picograms to hundreds of nanograms. Sex determination was based on previous studies validating testosterone concentrations in male and female green turtles (Owens 1997).

### Statistics

All statistical analyses were performed using JMP 4.02 (SAS Institute Inc. Cary, NC). No health parameters varied with either straight carapace length or sex, so all size classes and sexes were pooled for statistical analyses. A backward stepwise regression (*n* = 28) was run to analyze the relationship between blood Hg concentration and hematocrit, glucose, lymphocyte cell count, heterophil cell count, urea nitrogen, total protein, albumin, aspartate amino-transferase (AST), calcium, phosphorus, sodium, potassium, chlorine, globulin, creatine phosphokinase (CPK), and uric acid. The stepwise model used natural log-transformed Hg concentrations and was set to reject parameters with a significance level of *p* > 0.1. Relationships between blood Hg concentrations and *ex vivo* lymphocyte proliferation, lysozyme, and corticosterone were determined using the Spearman rho rank correlation test for the 2003 data set. The Pearson correlation test was used with log-transformed Hg concentration and lysozyme for the preliminary data from 2001 (*n* = 12). *In vitro* MeHg exposures were assessed using a two-way ANOVA (*p* < 0.05) with log-transformed SI, MeHg dose level, and turtle identification number (to account for variability in sensitivity and endogenous natural Hg concentrations among individuals). Each dose level was compared with the control using a pair-wise orthogonal *t*-test. The *in vitro* proliferative dose response was assessed further by regression analysis. The log transformation of the end point (SI) was plotted against the log dose. The log dose needed to achieve 50% suppression of the immune response was determined from the regression equation, and the inverse log was calculated to determine the ED_50_ (effective dose to produce a 50% response).

## Results

### THg concentrations

The samples from 2003 (*n* = 66) represent previously unpublished Hg analyses and range from 0.006 to 0.077 μg/g, with a mean (± 1 SE) Hg concentration of 0.029 ± 0.002 μg/g. This mean value is nearly identical to previously published blood Hg concentrations from loggerheads in the same region (Table 1). In a previous study (Day et al. 2005), blood Hg concentrations ranged from 0.005 to 0.188 μg/g, with a mean (± 1 SD) of 0.029 ± 0.008 μg/g (*n* = 34). The turtles in that study were captured in 2001 as part of the same sampling program and, where complementary data were available, were incorporated into comparisons to health parameters in the present study (Table 1). The consistency of these blood Hg values over a 3-year period suggests that these data are very representative of this population of loggerheads. The most notable difference in the Hg levels in these two data sets is the higher range in the 2001 samples compared with those from 2003.

### Clinical blood parameters

**Figure 2:**
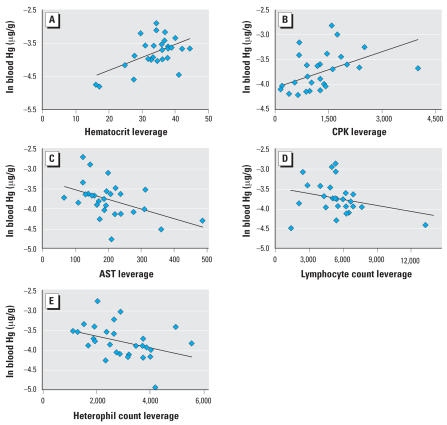
Natural log of blood Hg concentration (μg/g) versus clinical blood parameters for loggerhead sea turtles. Leverage plots from a multiple regression (*r*^2^ = 0.54; *p* = 0.003) show the relationship of blood Hg levels to hematocrit (*A; p* = 0.01), CPK (*B; p* = 0.03), AST (*C; p* = 0.02), lymphocyte cell count (*D; p* = 0.05), and heterophil cell count (*E; p* = 0.07). Four of the five health parameters varied significantly (*p* < 0.05) with blood Hg concentration. No health parameters varied with either age (indicated by straight carapace length) or sex, so all size classes and sexes were pooled for statistical analysis.

Summary statistics for blood chemistry and differential WBC counts (CBC) for these individuals are presented in Table 2. A comprehensive account of these parameters from the larger data set from which this subsample was taken was published by Maier et al. (2004). In the present study, all clinical blood parameters except hematocrit, CPK, lymphocyte cell count, heterophil cell count, and AST were rejected from the stepwise regression model (*r*^2^ = 0.54; *p* = 0.003). We observed a significant positive correlation between blood Hg and hematocrit (*p* = 0.01; Figure 2A) and CPK (*p* = 0.03; Figure 2B), and a negative correlation between blood Hg and AST (*p* = 0.02; Figure 2C). Within the differential WBC counts, we observed a significant negative correlation between blood Hg concentration and lymphocyte cell count (*p* = 0.05; Figure 2D).

### Ex vivo* lymphocyte proliferation as a function of natural THg exposure*

**Figure 3:**
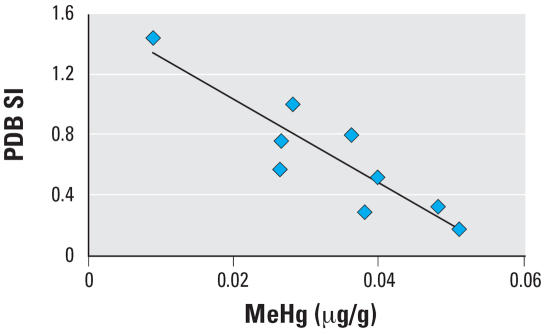
Linear regression between the endogenous blood Hg concentration (μg/g) and B-cell SI for the nine individuals in the *in vitro* control group for which Hg data were available. Samples were stimulated with PDB and incubated for 120 hr, with exposure to only the natural background Hg present in the blood, making this in essence another *ex vivo* proliferation assay. There was a highly significant negative relationship (*r*^2^ = 0.80; *p* = 0.001) across the natural range of Hg concentrations measured in these animals (0.009–0.051 μg/g).

A preliminary study using 12 samples collected during the summer of 2001 (Day 2003) indicated that PDB-induced B-cell proliferation was lower in loggerheads with higher blood Hg concentrations when incubated for 120 hr (Spearman ρ = –0.685; *p* = 0.014). Incubation times of 96 hr did not yield significant correlations in 2001. When repeated with the larger data set (*n* = 58) in 2003, the SI for T-cell and B-cell proliferation were not significantly correlated with blood Hg concentrations (*p* > 0.05) with incubation times of 96 hr. Because of technical difficulties, results from the 120-hr incubations were not available for the 2003 *ex vivo* assays. In addition to these planned *ex vivo* experiments, the results from another proxy *ex vivo* experiment are reported here. No MeHg dose was added to the control samples in the *in vitro* exposure, meaning that they were incubated for 120 hr with exposure to only the natural, endogenous Hg present in the blood. Therefore the *in vitro* control group is in essence another *ex vivo* proliferation assay. A linear regression between endogenous blood Hg concentrations and B-cell SI for the nine individuals for which Hg data were available showed a highly significant negative correlation (*r*^2^ = 0.80; *p* = 0.001) (Figure 3). The mean ± SE of SI, sample size, correlation coefficients, and *p*-value for these experiments are reported in Table 3 for each data set.

### Lysozyme activity

Assays performed on samples from 2001 (*n* = 12) showed a significant positive correlation between lysozyme activity and blood Hg concentrations (*r* = 0.63, *p* = 0.03) (Table 3). This data set was expanded using samples collected in 2003 (*n* = 58); however, lysozyme activity did not exhibit a significant relationship with blood Hg in the second set of experiments (*p* > 0.05). The mean absorbance for lysozyme activity, sample size, correlation coefficient, and *p*-value are reported in Table 3 for each data set.

### In vitro* proliferation following laboratory exposure to MeHg*

**Figure 4:**
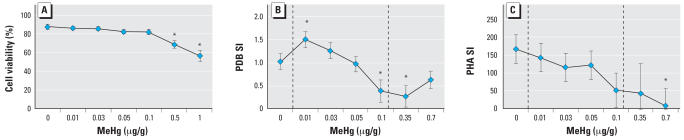
Loggerhead sea turtle lymphocyte proliferation following MeHg exposure. (*A*) Cell viability as measured with trypan blue (*n* = 8). (*B*) B-Cell proliferation stimulated with PDB (*n* = 25). (*C*) T-Cell proliferation stimulated with PHA (*n* = 10). Data are presented as mean ± SE. Vertical lines in (*B*) and (*C*) indicate maximum/minimum levels of total Hg previously measured in loggerhead sea turtle whole blood samples. *Significantly different from control (*p* < 0.05).

Cell viability was assessed by trypan blue at the termination of the exposure study. Treatment with 0.5 and 1 μg/g MeHg resulted in a significant decrease in cell viablity (Figure 4A). Statistical models for both B-cell and T-cell proliferation were highly significant (*p* < 0.0001) with *r* ^2^ = 0.72 and *r* ^2^ = 0.74, respectively. The dose level was a significant factor in both the B-cell (*p* < 0.0001) and T-cell (*p* < 0.01) models, as was the turtle identification number (*p* < 0.0001). B-cell proliferation (PDB-induced) was suppressed compared with the control at 0.1 μg/g (*p* = 0.02) and 0.35 μg/g (*p* < 0.0001), and enhanced relative to control at 0.01 μg/g (*p* = 0.04) (Figure 4B). The no observed effect level (NOEL) for suppression of B-cell proliferation was 0.05 μg/g Hg. The calculated ED_50_ for this response was 0.08 μg/g (*y* = 0.9891*x*^3^ + 3.5507*x*^2^ + 3.1556*x* + 0.2005; *r* ^2^ = 0.99). T-cell proliferation (PHA-induced) was suppressed at concentrations of 0.7 μg/g (*p* = 0.003; Figure 4C). The NOEL for T-cell proliferation was 0.35 μg/g Hg. The calculated ED_50_ for this response was 0.1 μg/g (*y* = –0.5244*x*^3^ – 2.1452*x*^2^ – 3.0577*x* + 0.482; *r* ^2^ = 0.93).

## Discussion

The use of blood to assess Hg exposure is an attractive option for studies on both human and wildlife health because blood can be non-lethally collected and allows for the simultaneous determination of clinical parameters that could be affected by Hg exposure. Blood is also an economical matrix for analysis because nearly all of the THg present in blood is MeHg (Henny et al. 2002), reducing the need for costly and time-consuming speciation. Therefore, the discussions of THg exposure levels *in vivo* and MeHg exposures *in vitro* are interchangeable for the purposes required here. This study represents the first published research investigating the relationship of Hg to health parameters in sea turtles. Overall, the blood Hg concentrations reported here are very similar to previous findings from the same population (Table 1; Day et al. 2005). Blood Hg concentrations in this population are slightly higher than those reported in loggerheads and Kemp’s ridleys (*Lepidochelys kempi*) from the Gulf of Mexico (Table 1) (Orvik 1997; Presti 1999; Wang 2003). Sea turtle blood Hg levels in general are lower compared with those of most marine mammals and seabirds, but the toxicologic significance of the blood Hg concentrations documented in most of these wildlife species remains unclear.

The positive relationship observed between blood Hg and hematocrit (Figure 2A) is consistent with the role of red blood cells (RBCs) as the primary transport mechanism of Hg throughout the body. Depending on the species, the cellular component of blood may have Hg concentrations 10–200 times higher than those of plasma because of the higher affinity of Hg for RBCs (Ansari et al. 1973; Kershaw et al. 1980; Magos 1987; Suzuki et al. 1971; U.S. EPA 1997). The Hg levels in these two compartments attain a relatively rapid equilibrium (Rabenstein et al. 1982, 1986), so the analysis of Hg in blood or plasma separately could provide a value representative of whole blood if a conversion factor was established for the organism of interest and the hematocrit was measured. In the absence of this information, it may be useful to analyze only RBCs for Hg, rather than using whole blood. This approach allows for an unbiased comparison among individuals with a wide range of hematocrit without the potential need for normalization, and also eliminates the impact of incidental dilution with other bodily fluids (e.g., lymph) during collection of the blood sample. Incidental collection of lymph is not typically a problem with sea turtles, but collecting blood from smaller animals with much smaller vessels and sinuses can prove more challenging. This approach also makes more efficient use of the collected blood volume by using only the portion of the blood where Hg is most concentrated, while leaving the plasma for other clinical parameters or organic contaminant analysis. This can be important when blood volume is a limiting factor because of health concerns or small body size, or when multiple analyses are required.

If the hematocrit count is omitted from the multiple regression analysis, the overall *r* ^2^ and *p*-values for the model change from 0.54 to 0.34 (*r*^2^) and from 0.003 to 0.04 (*p*), and CPK becomes the only parameter that is significant at α = 0.05. This demonstrates that including this covariate in the statistical analysis may have a beneficial impact on data interpretation, similar to using lipid-normalized concentrations for highly lipophilic organic contaminants. We observed this relationship between Hg and hematocrit in spite of the fact that most of the turtles in this sample set were fairly healthy and relatively few had hematocrit values far from the 36% that would be considered normal (Maier et al. 2004). Studies that include compromised animals in fields such as wildlife toxicology, heath assessments, or at rehabilitation centers could encounter more variable hematocrit counts and therefore an even greater potential for confounding effects. For example, “sick” loggerhead sea turtles captured in an abundance survey had an average hematocrit of 23% compared with 36% in healthy animals (Maier et al. 2004); in rehabilitation centers that receive chronically ill sea turtles, the hematocrit values are often < 10% (Norton TM, unpublished data). As suggested by Kershaw et al. (1980), if whole blood is analyzed for THg, hematocrit values should be obtained to determine if this could be a potential source of bias in the data.

CPK, an enzyme that is released into the blood from certain tissues when cellular damage occurs, is often used as an indicator of tissue damage (Evans 1996). In vertebrates, increased plasma CPK levels are typically associated with damage to skeletal muscle, heart, brain, or lungs (Evans 1996). The value of using CPK as an indicator of altered sea turtle physiology is currently unknown; however, if the interpretation in this taxon is consistent with other vertebrates, CPK levels suggest that individuals with higher blood Hg levels exhibit greater tissue damage. Based on mammalian studies, determination of which particular CPK isozyme is elevated would be necessary to identify the organ with which the cell damage is associated, or if any causal relationship with Hg exposure exists. Although not statistically significant, studies in both coturnix quail and European starlings fed Hg (HgCl or MeHg, respectively) for 12 weeks have shown a trend toward an increase in plasma CPK levels over control (Dieter 1974, 1975). On the basis of these previous studies, it is not unfounded that CPK was increased in relation to blood Hg in loggerhead turtles in the present study (Figure 2B).

Elevation of the enzyme AST is another indicator commonly used in clinical studies. Although AST is most commonly used to assess liver damage, it is also found in skeletal muscle, heart, pancreas, and kidneys (Evans 1996). AST may become elevated in response to various forms of stress in several different taxa, and most of the literature indicates that Hg exposure may also elevate plasma AST levels (Adachi et al. 1996; Hoffman et al. 2005). Relatively little is known about the distribution and activity of AST in sea turtles, but studies have found sea turtles with fibropapillomatosis have either elevated AST relative to disease-free animals (Aguirre et al. 1995; Norton et al. 1990) or no difference in AST (Swimmer 2000). Positive correlations between concentrations of AST and those of several organochlorine contaminants have also been noted in the plasma of loggerhead sea turtles (Keller et al. 2004). The health assessment effort that provided the samples in the present study found comparable levels of plasma AST in healthy and sick loggerhead sea turtles (Maier et al. 2004). Contradictory to what would be expected based on the literature, in the present study we found that turtles with higher Hg had lower plasma AST levels. The implications of this negative correlation and the diagnostic potential of AST in sea turtles are currently unclear.

Few data are available on the effects of chronic MeHg exposure on WBCs in wildlife. Our results indicate that loggerheads with higher blood Hg concentrations had lower numbers of circulating lymphocytes (Figure 2D). Thaxton et al. (1974) observed a decrease in total WBC numbers in broiler cockerels exposed to HgCl through drinking water on posthatch days 1–42. This was primarily due to a decrease in circulating numbers of lymphocytes and heterophils. However, in a study in Kemp’s ridley sea turtles, Peden-Adams et al. (2003) found no correlation between blood Hg levels and total WBC counts or WBC differential counts, but they did report a negative relationship between Hg levels in scutes and the number of circulating eosinophils. Hg is known to inhibit RNA and DNA synthesis and arrest cell-cycle progression in B lymphocytes (Daum et al. 1993). It is, therefore, plausible that chronic low-level Hg exposure could suppress the abundance of lymphocytes or alter numbers of specific classes of WBCs. It is important to note that with correlative field studies such as this, factors other than Hg may be related to the aforementioned hematologic changes. Further work is required to confirm whether Hg is a causative factor in the observed WBC count alterations.

Extensive work clearly shows Hg has immunomodulatory effects on a variety of taxa (Moszczynski 1997; Zelikoff et al. 1994). Evidence suggests that Hg has immunosuppressive effects for most lymphocyte functions, and this inhibition is often accompanied by an increase in susceptibility to infectious agents or tumor cells (Moszczynski 1997). For example, exposure of mice to MeHg increased their susceptibility to herpes virus (Christensen et al. 1996; Ellermann-Eriksen et al. 1994).

The preliminary *ex vivo* proliferation assessment performed in 2001 found a significant negative correlation between B-cell proliferation and blood Hg concentration (*n* = 12) but not T-cell proliferation. When the sample size was subsequently expanded (*n* = 58) using samples collected in 2003, no significant correlations were found between B-cell or T-cell proliferations (Table 3). There are two possible reasons for this inconsistency in the B-cell proliferation results. First, the 2001 data had a higher blood Hg concentration range and a smaller overall sample size than the 2003 data. This may have increased the chances of observing statistically significant correlations between Hg and lymphocyte proliferation in the 2001 data because of stronger leveraging by the individuals with high Hg levels that subsequently showed the most notable correlation. Second, in 2001, B-cell proliferation (PDB-induced) correlated with THg only after a 120-hr incubation (but not at 96 hr) before the addition of tritium to the plates. In 2003 data, technical difficulties caused PDB-induced proliferation to be available from the 96-hr incubation but not from the 120-hr incubation.

The observed correlation in the 2001 *ex vivo* data prompted the *in vitro* MeHg exposure experiments. Suppression of B-cell proliferation was observed *in vitro,* corroborating the 2001 *ex vivo* results. In addition, closer inspection of the *in vitro* data showed another trend supporting the relationship between B-cell proliferation and Hg: We found a strong negative correlation between B-cell proliferation and endogenous blood Hg concentrations in the *in vitro* control samples. The immunosuppressive response was observed not only across the range of *in vitro* dose levels (0–0.7 μg/g), but also across the much smaller range of natural Hg concentrations measured in animals within the control treatment (0.009–0.051 μg/g) (Figure 3). Like the 2001 *ex vivo* assays that showed significant B-cell suppression, the incubation time for the *in vitro* control was 120 hr. This supports the hypothesis that nonoptimal assay performance for the 2003 samples could have explained the inconsistency discussed above. This speaks to the sensitivity of the assay and possibly to the sensitivity of the loggerhead sea turtle immune system. Therefore, in future *in vitro* assays, we recommend incorporating the endogenous Hg concentrations of all individuals into the statistical model to account for this potential source of variability within dose groups.

Similarly, studies with Kemp’s ridley sea turtles in relation to blood Hg levels showed significant negative correlations between Hg and T-cell proliferation (Peden-Adams et al. 2003). These effects in sea turtles occur at substantially lower concentrations than reported for other vertebrates, such as rats and humans (Moszczynski 1997; Zelikoff et al. 1994). This is true even when accounting for the presence of the endogenous Hg present in addition to the dosed MeHg. This suggests the sea turtle immune system may be more sensitive to Hg toxicity. The concentrations used in the *in vitro* exposure were selected based on the range of blood Hg levels measured in wild animals (0.005–0.188 μg/g). Of the 100 animals sampled from this population, 5% had blood Hg levels above the 0.05-μg/g level that represents the highest exposure with no observed effect. Therefore, the *in vitro* experiment suggests that the portion of the loggerhead population with the highest Hg exposure could experience some suppression of immune function.

Circulating lysozyme is a marker of pro-inflammatory responses, has antibacterial functions, and is a measure of innate immunity (Burton et al. 2002; Weeks et al. 1992). It is secreted by granulocytes upon entry of foreign bacteria and lyses gram-positive bacterial cells by degrading the cell wall (Balfry and Iwama 2004; Ito et al. 1997). In fish, poly-chlorinated biphenyls (PCBs) are known to exhibit varied effects on lysozyme activity. For example, female dab (*Limanda limanda* L.) exposed for 7 days to sediments spiked with PCBs showed no differences in serum lysozyme activity (Hutchinson et al. 2003). However, channel catfish (*Ictalurus punctatus*) exposed to PCB-126 (a planar PCB) exhibited suppressed plasma lysozyme levels (Burton et al. 2002). In loggerhead turtles 4,4′-DDE (dichlorodiphenyltrichloroethylene) has been shown to exhibit a negative relationship with lysozyme activity (Keller et al. 2006b). Baginski (1988) found that mercury chloride exposure enhanced lysozyme release, which is consistent with the significant positive correlation between lysozyme activity and blood Hg concentration observed in the 2001 data. However, we observed no correlation between lysozyme and Hg in the 2003 study. As mentioned above, this could be related to the smaller sample size and the presence of animals with the higher Hg concentrations observed in the 2001 data set that drove the observed trend.

The combined results from the correlative field study and the *in vitro* exposures suggest that even low levels of Hg (relative to what is found in other species) may relate to altered health parameters in loggerhead sea turtles. The overall health impact of the higher CPK, lower lymphocyte counts, and suppressed B-cell proliferation observed in the wild population is unclear. The captured loggerheads all appeared to be in otherwise generally good health based on a superficial examination. In the case of the leverage plots from the multiple regressions (Figure 2), the considerable variability in the health parameters relates to blood Hg concentrations primarily at the extreme ranges. Similarly, based on the concentration range used in the *in vitro* exposure, approximately 5% of the wild population has blood Hg levels that correspond to a significant decrease in lymphocyte proliferation observed *in vitro*. Although the health impacts of these trends could be considered marginal, a moderate upward shift in the natural range of Hg exposures for these animals could result in greater impacts at the population level. Additionally, the *ex vivo* correlations suggest an even higher sensitivity to Hg immunomodulation. Few data are currently available on blood Hg concentrations in sea turtles from other regions. However, it is likely that in areas such as Florida Bay and the Indian River Lagoon (Florida), which have elevated levels of environmental Hg (Ache et al. 2000; Cantillo et al. 1999; Trocine and Trefry 1996), there is also a higher range of exposure for loggerheads and other sea turtle species. Perhaps not by coincidence, sea turtles in these areas also have an unusually high incidence of the debilitating and often fatal disease fibropapillomatosis. Environmental stressors have been implicated in the expression of fibropapillomas, but no work has currently been performed linking Hg to the immunosuppression that is often observed in individuals suffering from this disease. To more thoroughly address the potential impacts of Hg pollution on the overall health and fitness of these endangered species, future work will target areas where disease incidence and Hg levels are high.

## Figures and Tables

**Table 1:** Sea turtle blood Hg concentrations (μg/g wet mass) in the present study and in previously published work.

Study	Species	Location	Collection year	Mean ± SE (range)	No.
Present study (all samples)	*Caretta caretta*	Southeast (USA)	2003	0.029 ± 0.002 (0.006–0.077)	66
Day et al. 2005	*Caretta caretta*	Southeast (USA)	2001	0.029 ± 0.008 (0.005–0.188)	34
Presti 1999	*Caretta caretta*	Texas (USA)	1997	0.014 (0.01–0.017)	3
Presti 1999	*Lepidochelys kempi*	Texas (USA)	1997	0.027 (0.005–0.087)	76
Orvik 1997	*Lepidochelys kempi*	Texas (USA)	1994/1995	0.018 (0.0005–0.067)	106
Wang 2005	*Lepidochelys kempi*	Southeast/Gulf of Mexico (USA)	2000–2002	0.016 ± 0.002 (0.0006–0.179)	106
Wang 2005	*Lepidochelys kempi*	Rancho Neuvo, Mexico	2002	0.069 ± 0.009 (0.013–0.145)	18

**Table 2:** Summary statistics for clinical blood parameters and their relationships to total blood Hg concentration based on a backward stepwise multiple regression.

Parameter	Mean ± SE	*p*-Value
Hematocrit (%)^a^	35.7 ± 1.1	↑ 0.01
Total protein (g/dL)	5.1 ± 0.2	0.40
Albumin (g/dL)	1.0 ± 0.1	0.66
Globulin (g/dL)	3.0 ± 0.2	0.43
Glucose (mg/dL)	95.0 ± 5.6	0.80
Urea nitrogen (mg/dL)	65.2 ± 5.2	0.59
Uric acid (mg/dL)	1.2 ± 0.1	0.34
AST (U/L)^a^	203.1 ± 17.3	↓ 0.02
CPK (U/L)^a^	1257.2 ± 170.7	↑ 0.03
Calcium (mg/dL)	7.3 ± 0.2	0.83
Phosphorus (mg/dL)	7.6 ± 0.3	0.93
Sodium (mEq/L)	153.1 ± 1.3	0.94
Potassium (mEq/L)	4.5 ± 0.1	0.84
Chlorine (mEq/L)	113.0 ± 1.8	0.80
Lymphocyte counts (10^3^/μL)^a^	6,180 ± 662	↓ 0.05
Heterophil counts (10^3^/μL)^a^	2,928 ± 230	0.07

Arrows denote a significant relationship (α < 0.05) and indicate a positive or negative correlation to blood Hg concentration. The overall model had an *r*^2^ = 0.54 and *p* = 0.003. Hg concentrations in samples used in this analysis include 6 samples reported by Day et al. (2005) and 22 additional samples from the present study (*n* = 28). Hg concentrations ranged from 0.006 μg/g to 0.077 μg/g Hg, with a mean ± SE of 0.027 μg/g ± 0.003 μg/g Hg.

a Parameters with *p* < 0.1 that were not rejected from the model.

**Table 3:** Spearman rho correlation coefficient and *p*-values for *ex vivo* lymphocyte proliferation, lysozyme activity, and corticosterone versus total blood Hg concentration.

Assay (mitogen)	Mean ± SE	No.	Year	Incubation (hr)	ρ	*p-*Value
T cell (Con A)	3.02 ± 0.08	13	2001	120	–0.15	0.649
T cell (Con A)	8.08 ± 2.56	10	2001	96	–0.07	0.865
B cell (LPS)	1.87 ± 0.32	12	2001	120	–0.27	0.391
B cell (LPS)	8.13 ± 10.24	9	2001	96	–0.03	0.932
B cell (PDB)^a^	4.28 ± 0.95	12	2001	120	–0.69	0.014
B cell (PDB)	2.82 ± 1.08	9	2001	96	–0.38	0.309
Lysozyme activity^a^,^b^	4.96 ± 0.60	12	2001	—	0.63	0.029
T cell (Con A)	36.24 ± 18.67	57	2003	120	–0.22	0.105
T cell (PHA)	115.70 ± 25.63	57	2003	120	–0.03	0.829
B cell (LPS)	2.17 ± 0.85	58	2003	96	0.06	0.642
B cell (PDB)	2.17 ± 0.55	58	2003	96	–0.08	0.544
B cell (PDB)^a^,^c^	0.66 ± 0.13	9	2003	120	–0.80	0.001
Lysozyme activity	11.02 ± 0.82	58	2003	—	0.12	0.388
Corticosterone	2.61 ± 0.39	37	2003	—	0.20	0.242

Mean values represent SI for lymphocyte proliferation, lysozyme activity (μg/μL HEL), and corticosterone (ng/mL). No health parameters varied with either straight carapace length or sex, so all size classes and sexes were pooled for statistical analyses. Mean ± SE (range) of blood Hg concentration for samples collected in 2001 (*n* = 13) and 2003 (*n* = 58) were 0.037 ± 0.013 μg/g (0.012–0.188 μg/g) and 0.029 ± 0.002 μg/g (0.006–0.077 μg/g), respectively.

a Parameters were statistically significant.

b Pearson correlation coefficient is reported for lysozyme activity versus log-transformed Hg concentration, which showed a positive correlation.

c These data are from the control group from the *in vitro* exposure; *r*^2^ and *p*-values are reported for a linear regression, which showed a negative relationship (Figure 3).
